# Novel Semi-Nested Real-Time PCR Assay Leveraging Extendable Blocking Probes for Improved *SHOX2* Methylation Analysis in Lung Cancer

**DOI:** 10.3390/biom14060729

**Published:** 2024-06-19

**Authors:** Ngoc Anh Phuong, Trang Thuy Dao, Phuong Bich Pham, Ung Dinh Nguyen, Ba Van Nguyen, Tho Huu Ho

**Affiliations:** 1 Vietnam National Lung Hospital, 463 Hoang Hoa Tham, Ba Dinh, Hanoi 10000, Vietnam; phuongngocanhhmu@gmail.com; 2 Department of Genomics and Cytogenetics, Institute of Biomedicine and Pharmacy (IBP), Vietnam Military Medical University, No. 222 Phung Hung, Ha Dong, Hanoi 10000, Vietnam; 3 Oncology Center, 103 Military Hospital, Vietnam Military Medical University, Hanoi 10000, Vietnam

**Keywords:** lung cancer, *SHOX2* methylation, real-time PCR, semi-nested PCR, extendable blocking probes, DNA methylation, early detection

## Abstract

Lung cancer is the leading cause of cancer deaths globally, necessitating effective early detection methods. Traditional diagnostics like low-dose computed tomography (LDCT) often yield high false positive rates. *SHOX2* gene methylation has emerged as a promising biomarker. This study aimed to develop and validate a novel semi-nested real-time PCR assay enhancing sensitivity and specificity for detecting *SHOX2* methylation using extendable blocking probes (ExBPs). The assay integrates a semi-nested PCR approach with ExBPs, enhancing the detection of low-abundance methylated *SHOX2* DNA amidst unmethylated sequences. It was tested on spiked samples with varied methylation levels and on clinical samples from lung cancer patients and individuals with benign lung conditions. The assay detected methylated *SHOX2* DNA down to 0.01%. Clinical evaluations confirmed its ability to effectively differentiate between lung cancer patients and those with benign conditions, demonstrating enhanced sensitivity and specificity. The use of ExBPs minimized non-target sequence amplification, crucial for reducing false positives. The novel semi-nested real-time PCR assay offers a cost-effective, highly sensitive, and specific method for detecting *SHOX2* methylation, enhancing early lung cancer detection and monitoring, particularly valuable in resource-limited settings.

## 1. Introduction

Lung cancer, the most common cancer globally, accounted for 12.4% of all new diagnoses in 2022 and caused approximately 1.8 million deaths, making it the most lethal cancer by a considerable margin [1]. In Vietnam, a country particularly affected due to poor air quality in its megacities, lung cancer stands as the second most prevalent cancer following liver cancer, showing a troubling increase in incidence from 8900 cases in 2000 to 23,677 in 2018 [2].

Lung cancer primarily manifests in two forms: non-small cell lung cancer (NSCLC), which constitutes about 85% of cases, and the more aggressive small cell lung cancer (SCLC), which accounts for the remaining 15% [3]. The prognosis for lung cancer varies dramatically across stages; for instance, the 1-year survival rate for patients diagnosed at stage I (81–85%) is about five times higher than for those diagnosed at stage IV (15–19%) [4,5]. This disparity underscores the urgent need for early detection to improve survival outcomes.

Despite advancements, traditional screening methods such as low-dose computed tomography (LDCT) suffer from limitations, including high rates of false positives, psychological stress, and overdiagnosis, which may lead to unnecessary medical interventions [6,7]. Recent advancements in molecular diagnostics, particularly the study of DNA methylation, a stable epigenetic alteration often present in the early stages of lung cancer, have shown promise for enhancing the specificity and sensitivity of lung cancer detection [8,9].

*SHOX2*, a gene with extensive CpG methylation in its promoter region [10], has emerged as a potential biomarker for lung cancer [11]. The *SHOX2* gene comprises seven exons encoding a 319 amino acid protein with two large CpG islands. It has been characterized as a transcription factor involved in pattern formation [10]. The methylation of *SHOX2* has been associated with lung cancer development and has been studied in various biological materials, including plasma, where it demonstrated high specificity and sensitivity [10,12,13]. Building on these findings, this study introduces a novel semi-nested real-time PCR assay, designed to detect *SHOX2* methylation in plasma with enhanced sensitivity. This new methodology leverages an extendable blocking probe (ExBP) to improve the assay specificity, particularly in samples with low DNA concentrations. This assay aims not only to supplement but to refine the diagnostic accuracy, potentially reducing the reliance on invasive procedures and ambiguous imaging results.

The need to develop a high-quality, cost-effective method for detecting *SHOX2* methylation is critical, especially in developing countries where most reports to date have required expensive kits. This study’s findings are relevant not only for diagnostics but also for integrating molecular biomarkers with imaging techniques to advance lung cancer management. By providing a non-invasive, highly specific tool for early detection and ongoing patient monitoring, this research contributes to the evolving landscape of precision medicine in oncology.

## 2. Materials and Methods

### 2.1. Study Participants

The study recruited participants at Vietnam National Lung Hospital from August 2021 to November 2023 for two distinct sample collection groups to facilitate a comprehensive evaluation of the novel semi-nested real-time PCR assay for detecting *SHOX2* methylation. The first group provided fresh lung tissue biopsies and consisted of 12 individuals, including 9 patients diagnosed with lung cancer and 3 individuals with benign pulmonary diseases, as confirmed by histopathological evaluation. The second group, comprising 45 individuals, provided blood plasma samples. This group included 30 patients with lung cancer, categorized by disease stage: 15 patients ranged from stage IA to IIIA and another 15 extended from stage IIIB to IVB. The remaining 15 participants in this plasma group were diagnosed with non-cancerous pulmonary diseases. Table 1 below details the demographic and clinical characteristics of the participants providing tissue and blood plasma samples, delineating between those with lung cancer across various stages and those with non-cancerous pulmonary conditions.

This setup allowed the study to assess the assay’s efficacy across the different stages of lung cancer and compare findings against benign pulmonary conditions. Each participant was an adult aged (18 years or older) and provided complete demographic information, ensuring a robust framework for analyzing the results. This study was conducted according to the guidelines of the Declaration of Helsinki and was approved by the institutional research committee at Vietnam Military Medical University (reference number: 182/2021/CNChT-HĐĐĐ, dated 10 August 2021). All participants were thoroughly informed about the research content and objectives, and informed consent was obtained, ensuring voluntary participation and full awareness of the procedures involved.

Participants were selected based on specific inclusion criteria. For the group with lung cancer, participants had to have a confirmed diagnosis of lung cancer, be aged 18 years or older, provide complete information including administrative details, medical history, clinical signs, laboratory parameters, and imaging results, and consent to participate in this study. The control group included patients with non-cancerous lung lesions, evidenced by nodules, lung tumors, or consolidation on chest CT scans, with histopathologically confirmed non-cancerous lesions responding to non-specific treatments, or patients without biopsy but responding to non-specific treatments. These participants also had to be aged 18 years or older, provide complete information, and consent to participate in the study. Exclusion criteria included any cases that did not meet the inclusion criteria or patients who refused to cooperate during the study.

### 2.2. Sample Collection and Processing

Tissue and blood collection: Fresh lung tissue samples were collected from patients during surgical procedures and transported to the laboratory within 6 h, preserved in TRIzol™ Reagent (Thermo Fisher Scientific, Waltham, MA, USA). Approximately 10 mL of venous blood was drawn from each participant, using K2 EDTA tubes to prevent clotting. The blood samples were processed within 6 h to separate plasma, by centrifugation at 120× *g* for 20 min, which was then stored at −80 °C.

DNA extraction and quality assessment: DNA from plasma was extracted using the QIAamp Circulating Nucleic Acid Kit (Qiagen, Hilden, Germany). Similarly, lung tissue sections were processed to extract DNA with the QIAamp Tissue Kit (Qiagen, Hilden, Germany). The quantity and quality of extracted DNA were assessed using Nanodrop spectrophotometry.

Bisulfite conversion: Extracted DNA underwent bisulfite treatment using the EZ DNA Methylation-Gold™ Kit (Zymo Research, Tustin, CA, USA), converting unmethylated cytosine to uracil, which is then read as thymine during amplification, leaving the methylated cytosines unchanged.

### 2.3. Preparation of Standard DNA Samples

The preparation of standard samples for the development and validation of the novel semi-nested real-time PCR assay involved creating synthetic DNA calibrators that mimic the bisulfite-converted *SHOX2* gene sequences. These calibrators were synthesized to include either methylated or unmethylated versions of the *SHOX2* gene (Phusa Genomics, Can Tho, Vietnam). The unmethylated sequences underwent a bisulfite conversion where the CpG sites were transformed into TG, whereas the methylated sequences retained their original CpG sites.

To establish a range of methylation levels, a fixed concentration of unmethylated *SHOX2* DNA (10^6^ copies/µL) was mixed with varying concentrations of methylated *SHOX2* DNA. These varying concentrations were prepared to reflect methylation levels of 10%, 1%, and decreasing progressively to as low as 0.0001%, creating a gradient of methylation within the sample set. This methodical preparation allows for the precise quantification of the assay’s sensitivity to different levels of DNA methylation.

### 2.4. Semi-Nested Realtime PCR

The semi-nested real-time PCR assay designed for *SHOX2* methylation detection employs a two-stage amplification process to ensure high specificity and sensitivity. In the first stage, the reaction includes 1× HTOne MaX qPCR Green master mix (HT Biotec, Ho Chi Minh, Vietnam), forward primer (0.2 µM), reverse primer: (0.8 µM), and 5 µL template. The primers used in the first stage of the semi-nested real-time PCR assay include two specific sequences. The forward primer, SHOX2/FL, features the sequence CATACAACCCCAATCAAA-GTTTTTTGGATAGTTAGGTAAT, with the ExBP portion being underlined. This ExBP at the 5′ end of the forward primer is strategically designed to selectively enrich and preferentially amplify methylated DNA sequences. The reverse primer, SHOX2_Ro, has the sequence CCTCCTACCTTCTAACCC. These primers collectively target regions containing CpG sites, optimizing the detection of methylation within these critical genomic areas.

The first amplification stage is performed in an Applied Biosystems 9800 FAST Thermal Cycler (Thermo Fisher Scientific, USA) and begins with a denaturation step at 95 °C for 15 min. This is followed by four cycles of denaturation at 94 °C for 15 s, annealing at 50 °C for 60 s, and extension at 72 °C for 30 s. This sequence is then repeated for an additional 11 cycles, but with an extension occurring continuously at 72 °C. The amplified product from this first stage is diluted 20 times, and 5 µL of this diluted sample is used as the template for the subsequent round of amplification.

In the second stage of PCR, the reaction includes 1× HTOne MaX qPCR Probe master mix (HT Biotec, Ho Chi Minh, Vietnam), standard forward and reverse primers that do not target CpG sites, and a TaqMan probe specific for the methylated sequence. The forward primer is SHOX2_F (GTTTTTTGGATAGTTAGGTAAT), the reverse primer is SHOX2_Ri (AACCCTTTAAACAACCAAC), and the TaqMan probe is labeled with HEX (HEX-CTCGTACGACCCCGATCG-BHQ1). This setup allows for the detection of the fluorescent signal only when the probe hybridizes to its target methylated DNA, thereby confirming its presence.

The second amplification stage is conducted using the Rotor-Gene Q instrument (Qiagen, Germany) and includes an initial denaturation at 95 °C for 15 min, followed by 40 cycles at 94 °C for 15 s, 60 °C for 30 s, and 72 °C for 30 s.

Moreover, to quantify the total *SHOX2* gene levels, a parallel PCR reaction is conducted using the same primers and thermocycling program as the second stage but substitutes the TaqMan probe with SYBR Green. This reaction utilizes bisulfite-modified DNA that has not undergone amplification in the first stage as its template. This approach allows for an accurate measurement of the total *SHOX2* gene levels in the samples, providing an essential baseline for assessing the methylation levels relative to the overall DNA quantity. This dual approach ensures not only the detection of methylated DNA but also facilitates a detailed comparison of the methylation status across different samples, critical for accurate and reliable diagnostics in lung cancer.

### 2.5. Statistical Analysis

The statistical analysis was performed using MedCalc software version 20.019 (MedCalc Software Ltd., Ostend, Belgium). Data were presented as median and interquartile range or mean and standard deviation, depending on the distribution of the data. Appropriate tests such as the Mann–Whitney U test and the Chi-square test were employed to compare the differences between groups.

Receiver operating characteristic (ROC) curve analysis was utilized to evaluate the diagnostic value of the biomarkers under study. The area under the ROC curve (AUC) was calculated to assess the diagnostic accuracy, and optimal cut-off values were determined based on the ROC analysis.

## 3. Results and Discussion

### 3.1. Technical Overview of the Novel Semi-Nested Realtime PCR Assay for Detecting SHOX2 Methylation

**Figure 1:**
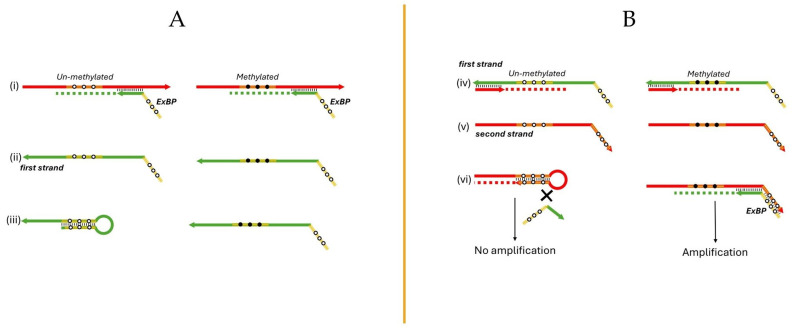
Schematic of the semi-nested real-time PCR assay with the ExBPs. (**A**) features extendable blocking probes (ExBPs) at the 5′ ends of forward primers (Panel (i)), which are designed to anneal to target regions free of CpG sites, allowing binding to both methylated and unmethylated sequences. Panel (ii) illustrates the synthesis of the ‘first strand’, initiated from the primer containing the ExBPs, with Panel (iii) depicting the specific hybridization of ExBPs to unmethylated CpG sites, and its inability to align with methylated sequences. (**B**) demonstrates the extension process of the PCR primer forming a complementary “second strand” from the first strand (Panels (iv) and (v)). Panel (vi) highlights the formation of a stable hairpin structure at the 3′ end of this second strand if derived from unmethylated DNA, effectively preventing it from acting as a template in subsequent PCR cycles/stages, and thereby enhancing the selective amplification of methylated DNA sequences.

The semi-nested real-time PCR assay incorporates innovative ExBPs to significantly enhance specificity in detecting methylated DNA. These ExBPs are strategically positioned at the 5′ ends of the forward PCR primers (Figure 1(Ai)). The forward and reverse primers themselves are designed to hybridize to regions that do not contain CpG sites, thus allowing them to anneal with both methylated and unmethylated sequences. This design facilitates the universal binding of the primers while the specificity of detecting methylated sequences is primarily driven by the action of the ExBPs.

The ExBPs are specifically designed to fully complement regions containing CpG sites on the newly synthesized DNA strand, which originates from the primer containing the ExBPs, and is referred to as the “first strand” (Figure 1(Aii)). These probes are designed to perfectly match the sequences of unmethylated DNA, but they do not completely match with methylated sequences, which is crucial for the assay’s selectivity (Figure 1(Aiii)). Upon the hybridization of a PCR primer to the mentioned first strand, it extends to form a complementary “second strand” (Figure 1(Biv,v)). If this second strand is derived from the unmethylated DNA, it can form a hairpin structure at its 3’ end. This hairpin undergoes primer extension to form a longer and more stable stem, which prevents the second strand from serving as a template in subsequent PCR cycles (Figure 1(Bvi)). This effectively inhibits the amplification of unmethylated DNA, thereby ensuring that only methylated DNA sequences are amplified, enhancing the assay’s discriminatory power and accuracy in molecular diagnostics.

In the second stage of our semi-nested real-time PCR assay, the TaqMan probe is engineered to bind specifically to methylated CpG sites, ensuring the detection of DNA methylation through fluorescence released upon probe cleavage by Taq polymerase. This assay uniquely incorporates the ExBPs that enhance specificity by selectively amplifying methylated DNA and suppressing the amplification of unmethylated sequences. This approach is crucial for distinguishing methylated DNA in samples predominantly containing unmethylated sequences. Furthermore, the assay’s semi-nested design optimizes sensitivity by conducting a two-round amplification process targeting three distinct primer-binding regions, thereby enhancing the detection of low-abundance methylated DNA, which is vital for early cancer diagnostics.

The newly developed semi-nested real-time PCR assay offers a streamlined approach to detecting *SHOX2* methylation, enhancing both specificity and cost-effectiveness, especially relevant in developing countries. Unlike the previously published methods that require complex and expensive modified probe designs, which are non-extendable, to differentiate between methylated and unmethylated DNA [12], the current method employs ExBPs, which is an integral part of the “unmodified” PCR primer itself. These ExBPs are designed to prevent the amplification of unmethylated DNA without the need for separate, intricate probe modifications. This not only reduces the cost and complexity of the assay but also minimizes the risk of generating non-specific PCR products, a common issue in highly sensitive assays that can lead to false positives. By focusing on simplifying the probe design while maintaining high assay sensitivity, this method effectively enriches for methylated sequences even in samples predominantly containing unmethylated DNA, making it an ideal tool for early cancer detection in a clinical setting.

Further validation with a more extensive array of sample types, including model DNA samples, fresh tissue biopsies, and blood samples from both lung cancer patients and controls, is crucial. These ongoing studies will provide comprehensive data to assess the assay’s practical applicability and diagnostic accuracy in a clinical environment, promising to refine parameters, enhance diagnostic precision, and establish the role of *SHOX2* methylation analysis revealed by the novel assay in precision oncology.

### 3.2. Evaluation of the Novel Assay Using Standard Samples

To validate the semi-nested PCR methodology developed for detecting *SHOX2* methylation, an extensive evaluation was conducted using synthetic DNA standards. These standards, designed to mimic the *SHOX2* gene sequence post-bisulfite modification, included both methylated and unmethylated forms. The experiment aimed to assess the assay’s sensitivity and specificity across a gradient of methylation levels, with DNA standards containing the varying proportions of methylated *SHOX2* ranging from 100% (fully methylated) down to 0.001%, and an additional unmethylated (0%) control.

**Figure 2:**
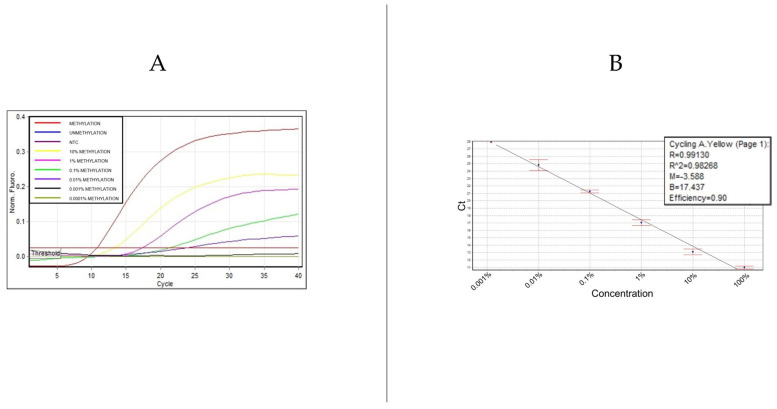
Amplification signals and standard curve analysis for *SHOX2* methylation detection. (**A**) illustrates amplification curves for *SHOX2* methylation levels from 100% down to 0.01%. Below 0.01%, amplification signals are detectable in one of two replicates. (**B**) presents the standard curve, displaying a linear relationship across a dynamic range of four logs, with a coefficient of determination (R^2^ = 0.98268) and an amplification efficiency of 0.90.

Results showed that the 100% methylated sample exhibited the strongest amplification signal, demonstrating the assay’s capability to detect high levels of methylation. Samples with decreasing concentrations of methylated DNA (10%, 1%, 0.1%, and 0.01%) still generated detectable signals, albeit with progressively lower intensities, highlighting the method’s sensitivity to even low levels of methylation. Crucially, the 0% methylated sample showed no amplification signal, confirming the assay’s specificity and its ability to effectively discriminate against unmethylated DNA (Figure 2).

The potential of this semi-nested PCR assay for precise methylation quantification is significant for early lung cancer detection and monitoring. We demonstrated a remarkable capacity to detect *SHOX2* methylation at levels as low as 0.01%. This sensitivity is significantly greater than that of traditional PCR methods and marks a substantial improvement over previously established assays, such as the HeavyMethyl technique [14]. While the HeavyMethyl assay detected methylated DNA in a background of unmethylated DNA at a ratio of 1:1000, corresponding to a 0.1% detection limit, our method pushes this limit to 0.01%. This ten-fold increase in sensitivity highlights the potential of the semi-nested real-time PCR approach to detect extremely low levels of methylation, which is crucial for early cancer detection and monitoring.

This enhanced detection capability is largely attributed to the strategic use of ExBPs in our assay, which selectively enriches methylated sequences by effectively blocking the amplification of unmethylated DNA. This specificity is critical in clinical settings, where the precise quantification of methylation levels can influence the diagnostic and therapeutic decisions for lung cancer patients.

This study lays a robust foundation for applying this assay to clinical samples in future phases, aiming to validate its diagnostic utility further. The integration of advanced molecular techniques, such as semi-nested real-time PCR with ExBPs, promises to enhance the accuracy of detecting crucial biomarkers like *SHOX2* methylation, setting the stage for the next generation of oncological diagnostic assays.

### 3.3. Testing on Fresh Tissue Samples from Lung Cancer and Benign Pulmonary Disease Patients

The results from the evaluation of the semi-nested real-time PCR assay on lung tissue biopsies are detailed below. This study aimed to identify the presence of *SHOX2* gene methylation in biopsy samples from a total of 12 patients, comprising nine lung cancer patients and three patients with benign lung conditions. The findings revealed that *SHOX2* methylation was detected in all biopsy samples from lung cancer patients, whereas no *SHOX2* methylation was observed in samples from individuals with benign lung conditions, as summarized in Table 2.

The PCR amplification signal provides a visual and precise means to detect *SHOX2* methylation, contributing to the efforts to diagnose and differentiate lung cancer from benign lung diseases. The results not only confirm the high specificity of *SHOX2* methylation in diagnosing lung cancer compared to benign conditions but also open new avenues for research into the use of this marker in blood sample analysis.

### 3.4. Evaluation on Plasma Samples from Lung Cancer and Benign Lung Disease Patients

Following the validation of the semi-nested real-time PCR assay on standard and tissue samples, the study extended its evaluation to plasma samples from patients with lung cancer (n = 30) and those with benign lung conditions (n = 15). This extension aimed to assess the assay’s practical application in a clinical setting where sample variability and DNA integrity are crucial factors.

In this phase, the effectiveness of the assay was gauged by analyzing delta Ct values, which denote the difference in cycle threshold (Ct) between the real-time PCR assay for methylation-specific detection and the total *SHOX2* gene detection. These delta Ct values are indicative of the proportion of *SHOX2* genes methylated relative to the total *SHOX2* genes present in the sample, providing a quantitative measure of methylation extent.

**Figure 3:**
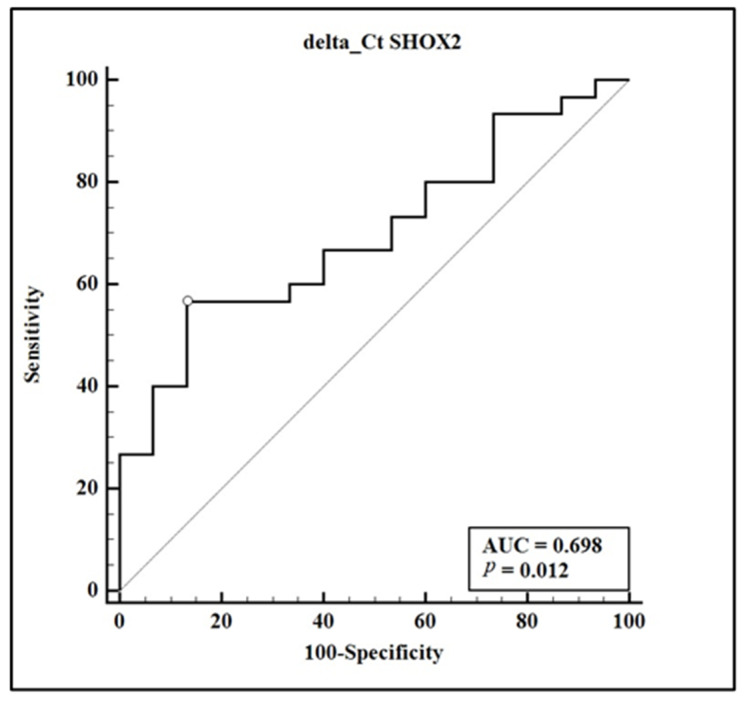
Receiver operating characteristic of *SHOX2* methylation in plasma. ROC curve displaying the diagnostic accuracy of *SHOX2* methylation levels in plasma to differentiate between lung cancer (n = 30) and benign lung disease (n = 15). The area under the curve (AUC) is 0.698, with a sensitivity of 56.67% and specificity of 86.67% at the optimal cut-off value of ≤11.855.

Statistical analysis revealed significant differences in delta Ct values between the cancer group and the benign group, with medians of 11.705 and 15.090, respectively, and a *p*-value of 0.0431 (Mann–Whitney test). This suggests that *SHOX2* methylation is notably higher among lung cancer patients. A receiver operating characteristic (ROC) curve was employed to further evaluate the diagnostic accuracy of these delta Ct values (Figure 3). The area under the curve (AUC) was 0.698, pointing to moderate diagnostic utility. Applying a cut-off value of ≤11.855 resulted in a sensitivity of 56.67% and a specificity of 86.67%, highlighting the assay’s capacity to distinguish between malignant and non-malignant conditions.

A chi-squared test was conducted to assess the difference in disease presence based on the delta Ct cutoff of 11.855, which revealed a significant association (chi-squared = 7.526, df = 1, *p* = 0.0061) with an odds ratio of 8.50 (95% CI: 1.62 to 44.46), emphasizing a robust correlation between lower delta Ct values and the likelihood of lung cancer.

Our study’s results align with previous findings, where *SHOX2* DNA methylation was validated as a robust biomarker for lung cancer detection in plasma samples [12]. Both studies observed that *SHOX2* methylation could effectively distinguish between malignant and non-malignant lung conditions. However, our semi-nested real-time PCR assay demonstrated an AUC of 0.698, which, while indicative of moderate diagnostic utility, is slightly lower than the AUC of 0.78 reported in the larger cohort of the referenced study. Notably, our assay achieved a sensitivity of 56.67% and a specificity of 86.67%, compared to the 60% sensitivity and 90% specificity reported in the mentioned study.

Moreover, our study highlights the potential of the assay in clinical applications not just for diagnosis but also for monitoring due to the enhanced sensitivity of the semi-nested approach for detecting low levels of methylated DNA. This aspect was not explicitly explored in the referenced study, which focused more on diagnostic efficacy. Additionally, our research employed a newly optimized semi-nested PCR technique that could potentially reduce costs and improve the accessibility of *SHOX2* methylation testing, an advantage not discussed in the earlier study.

The results from this assessment emphasize the potential of *SHOX2* methylation as a biomarker in plasma samples for lung cancer. The enhanced sensitivity of the semi-nested real-time PCR assay for detecting low levels of methylated DNA supports its use in early cancer detection and monitoring, crucial for improving patient prognosis.

The study’s limited sample size, while indicative of preliminary potential, underscores the necessity for further research with a broader sample base to validate these findings conclusively. The primary focus of this initial study was the development and testing of a new semi-nested real-time PCR assay across various sample types, including plasma. The promising results obtained from this diverse set of samples highlight the assay’s potential for accurate methylation detection. However, they also point to the need for extensive evaluations involving larger cohorts to establish the robustness and reliability of the assay in clinical settings.

Future research should prioritize expanding the sample size and diversity to include a wider range of clinical conditions and demographic variables. This expansion will help to assess the assay’s effectiveness across a broader spectrum of lung cancer patients and potentially other cancers where *SHOX2* methylation could serve as a biomarker. Such studies will be crucial in confirming the utility of this assay for routine clinical use, particularly in early cancer detection and monitoring, which are vital for improving patient outcomes in lung cancer treatment.

By systematically building on this foundational research, subsequent studies can refine the assay parameters, enhance diagnostic accuracy, and solidify the role of *SHOX2* methylation analysis in the landscape of precision oncology.

## 4. Conclusions

This study introduces a novel semi-nested real-time PCR assay using extendable blocking probes (ExBPs) that enhance the detection of *SHOX2* methylation, demonstrating high sensitivity and specificity in distinguishing between lung cancer and benign pulmonary conditions. The innovative use of ExBPs not only improves assay accuracy but also significantly reduces costs by eliminating the need for complex probe modifications. This method achieves a high degree of sensitivity, capable of detecting lower concentrations of methylation compared to previous methods, thus facilitating early lung cancer detection and potentially reducing reliance on invasive diagnostics. Although the initial results are promising, further validation with larger and more diverse cohorts is essential to confirm the assay’s utility in routine clinical practice. This advancement represents a significant step forward in precision oncology, offering new possibilities for the early detection and management of lung cancer, especially in resource-limited settings.

## Figures and Tables

**Table 1:** Demographic and clinical characteristics of the study participants.

**Plasma Sample Donors (n = 45):**					
**Diagnosis**	LC	NC	**Sex**	LC	NC
Tuberculosis		8	Male	21	13
Pneumonia		2	Female	9	2
Pulmonary fungal infection		5			
Lung cancer	30		**Cancer stage**		
**Age (years)**			I	8	
<55	5	7	II	2	
55–70	21	6	III	6	
≥70	4	2	IV	14	
**Tissue Sample Donors (n = 12):**					
**Diagnosis**	LC	NC	**Sex**	LC	NC
Tuberculosis		0	Male	5	1
Pneumonia		2	Female	4	2
Spindle cell oriented fibroma		1			
Lung cancer	9		**Cancer stage**		
**Age (years)**			I	0	
<55	2	2	II	0	
55–70	6	0	III	3	
≥70	1	1	IV	6	

LC = Lung cancer; NC = Non cancer.

**Table 2:** Analysis of *SHOX2* methylation in lung tissue biopsies.

S.No	Patient ID	Patient Group	*SHOX2* Methylation Detection
1	BN001; 003	Non-small cell carcinoma	Positive
2	BN002; 006; 007; 011	Adenocarcinoma	Positive
3	BN004	Spindle cell-oriented fibroma	Negative
4	BN005	Low cellularity carcinoma	Positive
5	BN009	Pneumonia with bacterial superinfection	Negative
6	BN010	Chronic fibrotic lung tissue	Negative
7	BN012	Mixed non-small cell and squamous carcinoma	Positive

## Data Availability

The original contributions presented in the study are included in the article, further inquiries can be directed to the corresponding author.
